# An Illustrated Encyclopœdic Medical Dictionary

**Published:** 1888-08

**Authors:** 


### Book Reviews.

**An Illustrated Encyclopœdic Medical Dictionary.** Being a dictionary of the technical terms used by writers on medicine and the collateral sciences, in the Latin, English, French and German languages. By Frank P. Foster, M. D., editor of the *New York Medical Journal*, assisted by eleven collaborators. Vol. I., with illustrations. 4to, pp. xii., 752. New York: D. Appleton & Co. 1888.

The vocabulary of medicine has become so voluminous of late with its increased nomenclature in all its various branches, as to outgrow all the explanatory word-books extant. When

Dunglison, who has been since 1839 acknowledged authority as a medical lexicographer, published the fourth edition of his dictionary, it was embraced in an octavo volume of 771 pages, and claimed, not without justice, to be a work "in which the student may search, without disappointment, for every term that has been legitimated in the nomenclature of the science."

The first volume of Foster's work, a quarto, contains 752 pages, reaching from A to Cacos only. From this crude comparison some idea may be approximatively obtained as to the enormous growth of medical nomenclature and terminology in the last fifty years.

Up to the present time no adequate dictionary of medical terms has appeared to meet the requirements of the ever-advancing science. CAMPBELL'S *Language of Medicine* supplies the student and teacher with a great right arm, in attempting to master the origin and construction of medical technology; but its scope is limited as compared with Foster's encyclopœdic work; and, indeed, it is of a nature so different as to hardly permit of comparison with it.

The enormous labor involved in the preparation of the work under consideration is difficult to appreciate, and almost impossible to estimate. Begun in 1881, it has absorbed all the time the editor could possibly devote to such labor down to his recent severe illness, and it is greatly to be feared that his indefatigable application to this arduous duty has had much to do with precipitating the well-nigh fatal malady.

Besides the work bestowed upon it by Dr. Foster, he has been ably assisted by Drs. Ayres, Bronson, Bull, Coe, Currier, Duane, Garrigues, Kelsey, Norris, Wilder and Mr. Gage—all able, accomplished and wisely-chosen co-adjutors—scholars in the broadest sense.

The editor truly says in his preface: "The science in the present age is recorded in no one language; to learn it, one must at least read its exposition in English, French and German." This is at once a plea and a demand for higher medical education. Let no person disregard it in contemplating entrance to the ranks of medicine; let no teacher ignore it in his zeal to obtain a large matriculant constituency.

It is impossible to adequately review such a comprehensive system of lexicography as Foster's in the narrow limits that may properly be allotted to its consideration in this JOURNAL; nor, indeed, is it necessary, for its first volume is a book that must be seen to be appreciated, and every physician who would do himself justice must possess it. If it be urged that it is too voluminous for a dictionary, and thus too expensive for the average physician, that is not the fault of the editor or publishers, but rather of the science itself, which demands such a vast vocabulary for its exposition. It is one of the growing evils of the literature of medicine that new words, endings, and compoundings are being constantly added by the thousands. It would be far better, to our mind, if every writer would endeavor to simplify and cut down his vocabulary, rather than to seek the evanescent and doubtful reputation of a mere maker of words.

There is so little about the volume to criticise, we may be pardoned for excepting to the method the author has chosen to credit the contributors with their work. If he had employed the initials of the collaborators for this purpose, numbering them when duplicates appeared (as $B^1$, $B^2$, etc.), it would have been less confusing. However, this affects only the contributors themselves, and not the value of the work to the purchaser.

One word concerning the publishers' part of the work: it is difficult to see how it could have been done better. The illustrations are artistic specimens of wood engraving, the press-work superb, and the paper No. 1, highly-finished book paper,—and altogether, the mechanical work of the volume a credit even to the celebrated house that issues it.

